# Presbyvestibulopathy: an uncommon cause of dizziness in the elderly

**DOI:** 10.3389/fneur.2026.1744462

**Published:** 2026-03-05

**Authors:** Tzu-Pu Chang, Yu-Hung Kuo, Anand K. Bery

**Affiliations:** 1 Department of Neurology, Neuro-Medical Scientific Center, Taichung Tzu Chi Hospital, Buddhist Tzu Chi Medical Foundation, Taichung, Taiwan; 2 Department of Neurology, School of Medicine, Tzu Chi University, Hualien, Taiwan; 3 Department of Research, Taichung Tzu Chi Hospital, Buddhist Tzu Chi Medical Foundation, Taichung, Taiwan; 4 Department of Otolaryngology, Harvard Medical School, Boston, MA, United States

**Keywords:** aging, dizziness, geriatrics, vertigo, vestibular function, vestibular loss, vestibular testing

## Abstract

**Background:**

Dizziness is common in older adults, and often presents a diagnostic challenge. Vestibular function declines with age, but the prevalence and diagnostic explanatory value of mild vestibular loss in isolation (i.e., the clinical diagnosis of “presbyvestibulopathy”) remains uncertain.

**Methods:**

We retrospectively analyzed consecutive patients aged ≥ 60 years in a subspecialty dizziness clinic who underwent clinical assessment (history, exam, videonystagmography) and horizontal canal video head impulse testing. We examined (i) the prevalence of vestibular loss (by laterality/degree of loss), including across age groups, and (ii) the distribution of primary diagnoses and proportion attributable to presbyvestibulopathy.

**Results:**

Of the 102 included patients (72% female, mean age 72.8 years), 70 (68.6%) had normal vestibular function. Seven (6.9%) had mild bilateral vestibular hypofunction (vestibulo-ocular reflex [VOR] gain of 0.6–0.8 bilaterally). A smaller number had more severe degrees of bilateral hypofunction: severe (*n* = 6), and asymmetric (*n* = 4). Right-sided VOR gain was highest in those aged 60–69 years (0.96), intermediate in those aged 70–79 years (0.89), and lowest in patients aged ≥ 80 years (0.77; *p* < 0.001). Presbyvestibulopathy was uncommon as a final diagnosis across all age groups, with a total prevalence of 2.9% (*n* = 3). All three of these patients had treatable comorbidities (e.g., anxiety).

**Conclusion:**

VOR gain by vHIT declines with age in patients with symptomatic dizziness; however, most patients aged ≥ 60 still demonstrate normal VOR gains. The diagnosis of presbyvestibulopathy is uncommon in older adults with dizziness; a more specific vestibular diagnosis is often more appropriate. Even in those with presbyvestibulopathy, treatment of comorbidities can improve symptoms and function.

## Introduction

Chronic dizziness in older adults presents a significant diagnostic challenge. Its clinical manifestations are often nonspecific, and difficult to ascribe to a single etiology. When one clear cause is not found, some experts attribute this condition to vestibular degeneration (1, 2). Indeed, the prevalence of dizziness is markedly higher in the elderly, affecting up to 30–45% of this population (3, 4), and compelling histological and physiological evidence demonstrates that both peripheral and central vestibular systems degenerate with age (5–7). Age-related vestibular loss not only reduces the vestibulo-ocular reflex (VOR), but also increases the threshold for vestibular perception, which may contribute to dizziness (8–10). In addition, degeneration of the central vestibular system and age-related differences in central compensation may further contribute to the chronicity of dizziness in the elderly (11–13). Accordingly, the International Classification of Vestibular Disorders (ICVD) from the Bárány Society defines *presbyvestibulopathy* as a disease entity to describe chronic dizziness caused by age-related vestibular decline (14).

According to the ICVD diagnostic criteria for presbyvestibulopathy, patients must be at least 60 years old and present with a chronic vestibular syndrome. Bilateral semicircular canal function should be mildly impaired—for example, VOR gain between 0.6 and 0.8 on the video head impulse test (vHIT) (Table 1). Severe impairment (e.g., VOR gain < 0.6 on vHIT) meets criteria for bilateral vestibulopathy, in which etiologies other than aging should be considered (15). We know from normative data that, unlike other measures of vestibular function, VOR gain by vHIT remains relatively unaffected by age, to roughly age 80–90 (16).

**Table 1:** ICVD diagnostic criteria of presbyvestibulopathy.

A. Chronic vestibular syndrome (at least 3 months duration) with at least 2 of the following symptoms: Postural imbalance or unsteadiness Gait disturbance Chronic dizziness Recurrent falls B. Mild bilateral peripheral vestibular hypofunction documented by at least 1 of the following: VOR gain measured by video head impulse test between 0.6 and 0.8 bilaterally VOR gain between 0.1 and 0.3 upon sinusoidal stimulation on a rotatory chair (0.1 Hz, Vmax = 50–60^°^/sec) Reduced caloric response (sum of bi-thermal maximum peak SPV on each side between 6 and 25^°^/sec) C. Age ≥ 60 years D. Not better accounted for by another disease or disorder

ICVD, International Classification of Vestibular Disorders; VOR, vestibulo-ocular reflex; Vmax, maximum velocity; SPV, slow-phase velocity.

However, the clinical applicability of presbyvestibulopathy and its diagnostic criteria remains uncertain. Older adults experience not only age-related vestibular decline but also age-related deterioration of visual and somatosensory systems, both of which are essential for maintaining balance (2). Some experts therefore propose the concept of “multisensory dizziness,” in which multiple sensory systems contribute to instability, making it difficult to determine the leading cause (17). Moreover, many studies suggest that dizziness in the elderly is often multifactorial, related to anxiety or depression, blood pressure fluctuations, cardiovascular disease, polypharmacy, gait disturbance, and other comorbidities (18, 19). This multifactorial nature complicates both diagnosis and management.

In recent years, some have proposed an association between unexplained dizziness in the elderly and cerebral small vessel disease (20). Furthermore, because persistent postural-perceptual dizziness (PPPD) is the most common cause of chronic dizziness overall, it remains unclear whether chronic dizziness in the elderly represents a variant of PPPD (21, 22).

Dizziness substantially impairs quality of life and increases the risk of falls in older adults, underscoring the importance of accurate diagnosis and appropriate management (23, 24). However, given the multiple potential contributing factors outlined above, it remains unclear whether dizziness in most older patients can be adequately explained by presbyvestibulopathy and its current diagnostic criteria, or whether it should instead be attributed to other age-related conditions such as multisensory or multifactorial dizziness and cerebral small vessel disease, or classified as a variant of common vestibular disorders such as PPPD.

Given these uncertainties, it is important to clarify how many cases of dizziness in older patients can be explained by presbyvestibulopathy (mild vestibular loss) in isolation. Therefore, this study aimed to determine the prevalence of presbyvestibulopathy, in elderly patients with dizziness, across different age groups.

## Methods

We retrospectively analyzed consecutive patients aged ≥ 60 years who presented with dizziness or vertigo at our Neurology Dizziness Clinic in Taichung Tzu Chi Hospital (a regional hospital affiliated with Tzu Chi University) between November 2024 and June 2025. The patients routinely underwent a structured clinical history, neurological examination, and neuro-otological assessment. Bedside testing included video-nystagmography to evaluate spontaneous nystagmus with fixation block, skull vibration-induced nystagmus, head-shaking-induced nystagmus, and nystagmus during positional maneuvers (bow-and-lean, Dix–Hallpike, and supine-roll tests). Horizontal vHIT using the Synapsys Ulmer system (Marseille, France) was performed by a well-trained technician (SJW) with seven years of experience in vHIT operation.

We comprehensively reviewed the electronic medical records and vHIT data of consecutive patients presenting with dizziness in our clinic. Patients were included if they (i) were aged ≥ 60 years, (ii) completed a structured clinical history and clinical examinations, and (iii) underwent vHIT testing. Patients were excluded if they (i) did not complete the required clinical history and examinations or (ii) were unable to undergo vHIT testing due to cervical spine disease contraindicating high-frequency head impulses or other reasons. The study period (November 2024 to June 2025) was selected to ensure the inclusion of a sufficient number of patients in each age group (60–69, 70–79, and ≥80 years) for comparative analysis. This study was conducted in accordance with the Declaration of Helsinki and was approved by the Research Ethics Committee of Taichung Tzu Chi Hospital (REC 109–64).

During vHIT testing, each patient received 20 rightward and 20 leftward head impulses. Only head impulses with velocities between 100°/s and 300°/s were accepted for vHIT recordings. Vestibular hypofunction was defined as a VOR gain < 0.8 on vHIT. Patients were accordingly classified as having normal vestibular function, bilateral vestibular hypofunction (BVH), or unilateral vestibular hypofunction (UVH). BVH was further categorized as mild, severe, or asymmetric, while UVH was classified as mild or severe. Mild BVH was defined as a bilateral VOR gain of 0.6–0.8, consistent with the ICVD diagnostic criteria for presbyvestibulopathy (14). Severe BVH was defined as a bilateral VOR gain < 0.6, consistent with the ICVD diagnostic criteria for bilateral vestibulopathy (15). Asymmetric BVH was defined as a VOR gain of 0.6–0.8 on one side and < 0.6 on the other. Mild UVH was defined as a unilateral VOR gain of 0.6–0.8, while severe UVH was defined as a unilateral VOR gain < 0.6.

Based on clinical presentation, patients were classified into acute vestibular syndrome (AVS), episodic vestibular syndrome (EVS), or chronic vestibular syndrome (CVS). A well-trained otoneurologist (TPC) reviewed all clinical information and assigned a primary diagnosis. Common vestibular disorders—including vestibular migraine (VM) (25), benign paroxysmal positional vertigo (BPPV) (26), Ménière’s disease (27), vestibular neuritis (28), PPPD (29), mal de débarquement syndrome (30), vestibular paroxysmia (31), and bilateral vestibulopathy (15) — were diagnosed according to Bárány Society ICVD criteria. Other conditions were defined in the Appendix.

Only patients who (1) presented with CVS, (2) had bilateral VOR gain between 0.6 and 0.8 on vHIT, and (3) lacked any other primary diagnosis were classified as having a primary diagnosis of presbyvestibulopathy.

We analyzed (1) the prevalence of UVH, BVH (including mild and severe BVH) based on vHIT, and compared the VOR gains across age groups using a two-factor repeated-measures ANOVA; (2) the distribution of primary diagnoses and proportion attributable to presbyvestibulopathy; and (3) age-related prevalence of final diagnoses across groups (60–69, 70–79, ≥80 years). SPSS version 23 (Armonk, New York) was used for statistics.

## Results

### Demographic data

A total of 113 consecutive patients were initially enrolled. After excluding 11 patients who did not complete all examinations, 102 patients were included in the final analysis. Of these, 73 (71.6%) were female. The mean age was 72.8 years (range 60–92). Forty-one patients (40.2%) were aged 60–69 years, 32 (31.4%) were aged 70–79 years, and 29 (28.4%) were aged ≥ 80 years.

### Findings of the video head impulse test

**Figure 1:**
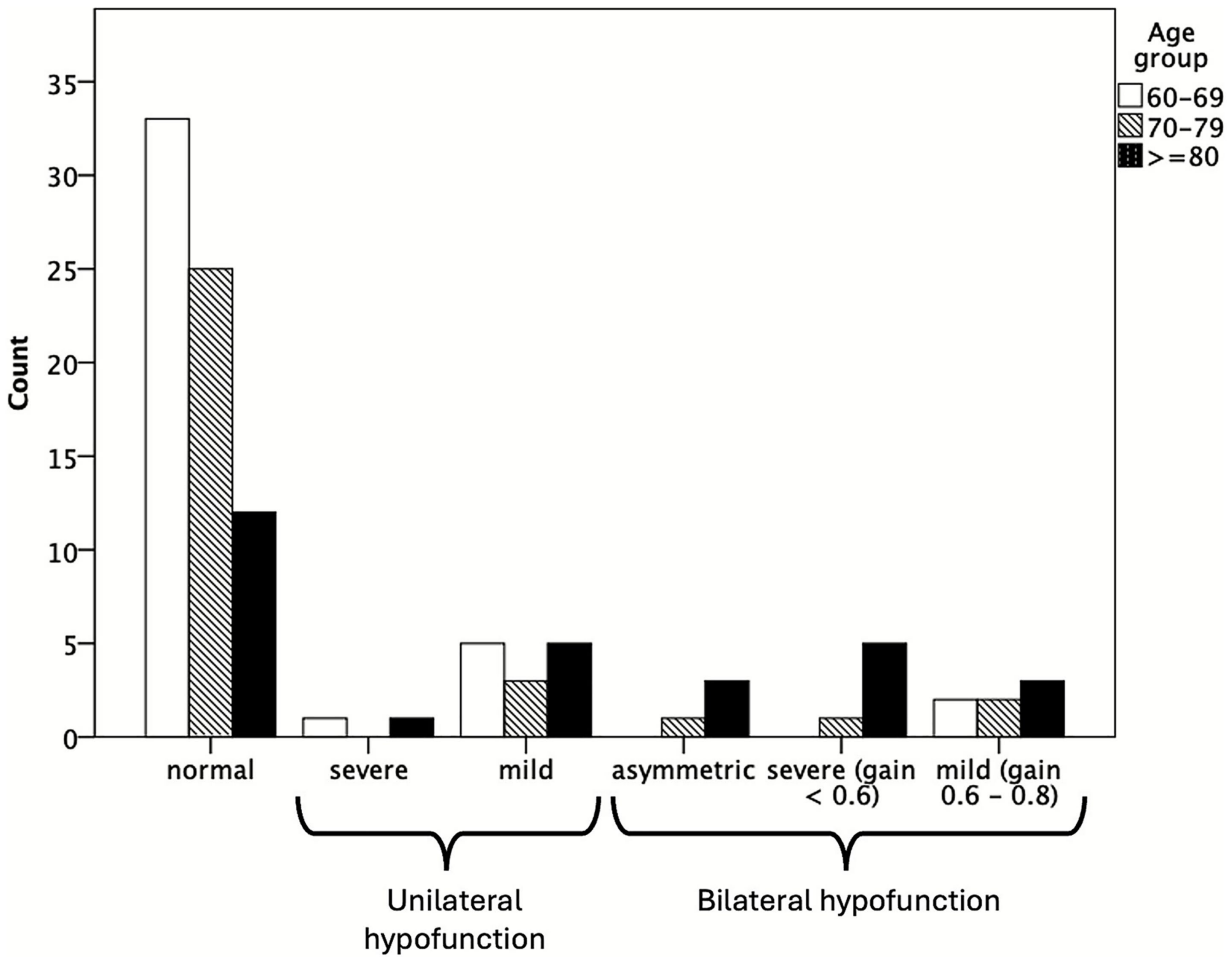
Distribution of vestibular hypofunction laterality and severity, regardless of final clinical diagnosis. *Mild bilateral vestibular hypofunction was defined as a bilateral VOR gain of 0.6–0.8, consistent with the diagnostic criteria for presbyvestibulopathy. The other groups are as defined in the Methods section.

Seventy patients (68.6%) showed normal vestibular function on vHIT, whereas 32 (31.4%) had vestibular hypofunction. Figure 1 illustrates the distribution of vestibular hypofunction laterality and severity. Seven patients (6.9%) had mild BVH, defined as a VOR gain of 0.6–0.8 bilaterally, consistent with the vHIT definition in the diagnostic criteria for presbyvestibulopathy. Six patients (5.9%) had severe BVH, with a VOR gain < 0.6 bilaterally, which met the vHIT definition for bilateral vestibulopathy. An additional four patients (3.9%) had asymmetric BVH, with one side showing a VOR gain of 0.6–0.8 and the other < 0.6.

**Figure 2:**
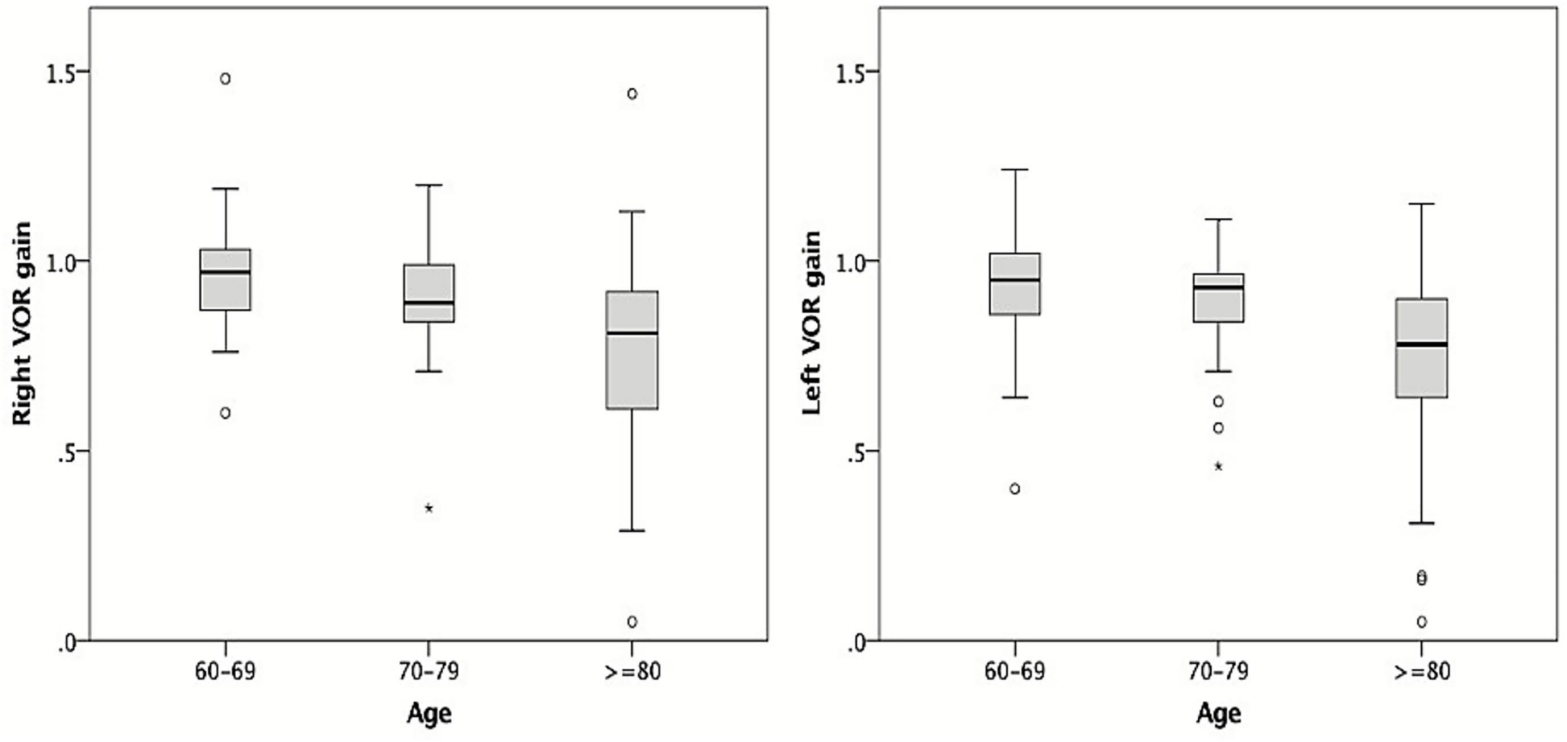
Comparison of the VOR gains of video head impulse test across age groups. The box-and-whisker plots show median (central line), upper and lower quartiles, upper and lower extremes, and outliers.

When stratified by age, right-sided VOR gain was highest in those aged 60–69 years (0.96, 95% CI 0.92–1.00), intermediate in those aged 70–79 years (0.89, 95% CI 0.84–0.94), and lowest in patients aged ≥ 80 years (0.77, 95% CI 0.66–0.88; *p* < 0.001). A similar trend was observed for the left-sided VOR gain (0.93 [95% CI 0.89–0.97] vs. 0.90 [95% CI 0.85–0.95] vs. 0.73 [95% CI 0.62–0.84]; *p* < 0.001) (Figure 2). The absolute difference was pronounced between the ≥ 80-year and 70–79-year groups but small between the 70–79-year and 60–69-year groups. On the other hand, there was no significant difference between right- and left-side VOR gain (*p* = 0.06), and no interaction was observed between age group and side (right vs. left) on VOR gain (*p* = 0.37).

### Primary diagnosis and comorbidities

Among the entire cohort, four patients (3.9%) presented with AVS, 56 (54.9%) with EVS, and 42 (41.1%) with CVS. A complete list of primary diagnoses is provided in the Supplementary Table 1. Table 2 summarizes the prevalence of the most common diagnoses, along with presbyvestibulopathy, across age groups. VM, BPPV, and PPPD were the leading diagnoses in patients aged 60–69 and 70–79 years. In contrast, bilateral vestibulopathy and Ménière’s disease were more frequent than PPPD and comparable in prevalence to VM among those aged ≥80 years. Presbyvestibulopathy was uncommon across all groups, with a prevalence of 2.4–3.4%.

**Table 2:** The prevalence of leading clinical diagnoses and presbyvestibulopathy among age groups.

Primary diagnosis	Total across all ages (% of total) *n* = 102	Age 60–69 (% of age group) *n* = 41	Age 70–79 (% of age group) *n* = 32	Age ≥ 80 (% of age group) *n* = 29	Mean VOR gain^*^
VM	21 (20.6)	12 (29.3)	7 (21.9)	4 (13.8)	0.95
BPPV	20 (19.6)	9 (22.0)	5 (15.6)	6 (20.7)	0.88
PPPD	15 (14.7)	7 (17.1)	5 (15.6)	1 (3.4)	0.97
Ménière’s disease	8 (7.8)	1 (2.4)	3 (9.4)	4 (13.8)	0.92
Bilateral vestibulopathy	4 (3.9)	0 (0)	0 (0)	4 (13.8)	0.32
Presbyvestibulopathy	3 (2.9)	1 (2.4)	1 (3.1)	1 (3.4)	0.74

VM, vestibular migraine; BPPV, Benign paroxysmal positional vertigo; PPPD, persistent postural-perceptual dizziness.

*Mean values of the averaged right and left VOR gains across individual patients.

**Figure 3:**
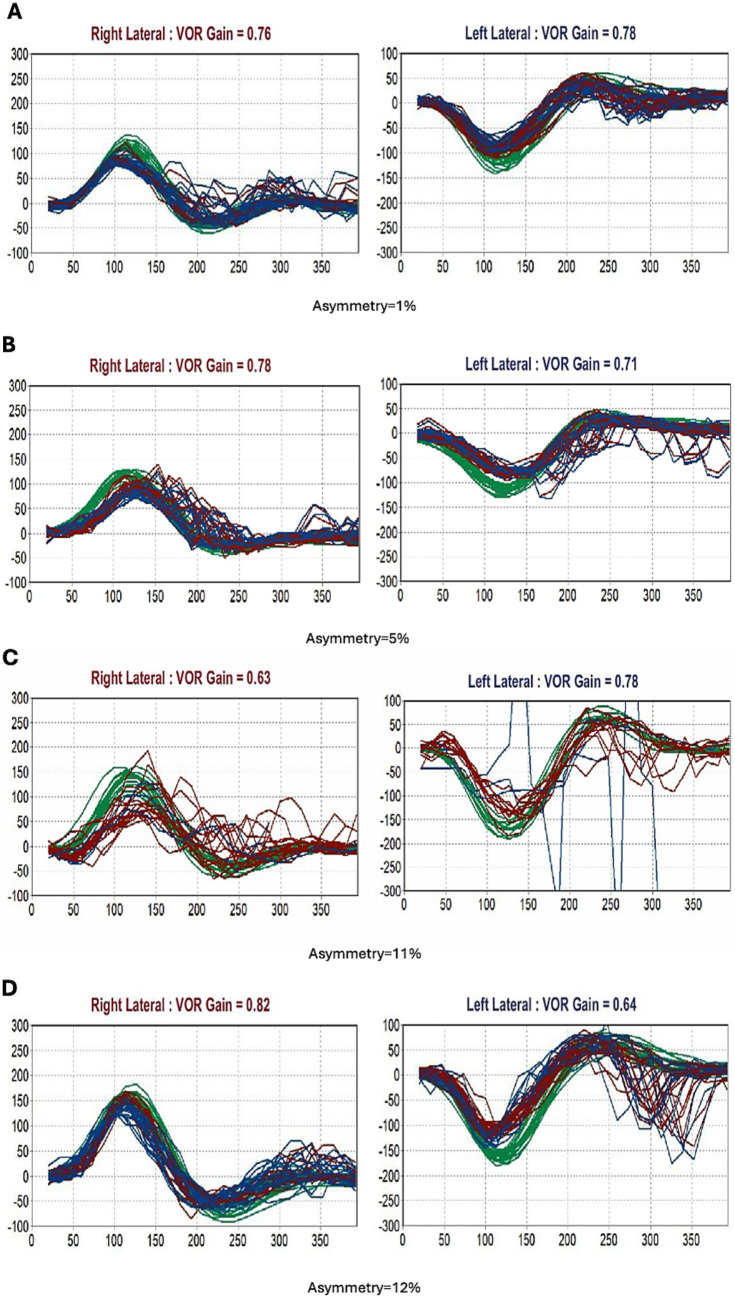
Video head impulse test findings in three patients with a primary diagnosis of presbyvestibulopathy [ages 69, 71, and 83 **(A–C)**] and one 88-year-old patient with chronic vestibular syndrome of uncertain primary diagnosis **(D)**. Green curves represent head velocity, purple right eye velocity, and blue left eye velocity; each trace corresponds to a single head impulse. In all cases, VOR gain was mildly reduced. In patients **(A–C)**, small, scattered catch-up saccades suggest mild bilateral vestibular hypofunction, which may be incidental and contribute less to dizziness perception in the elderly. In case **(C)**, the left eye signal was unobtainable due to cataract, with minor artifacts in the right eye trace. In case **(D)**, mild unilateral vestibular hypofunction with asymmetric gain reduction and moderate left-sided catch-up saccades raises the possibility of a contributory, though not definitive, role in the patient’s chronic dizziness.

Table 3 details the primary diagnoses and comorbidities of the seven patients with mild BVH (bilateral VOR gain 0.6–0.8). Note that only three of these patients had an ICVD final diagnosis of presbyvestibulopathy; for the other four, another vestibular diagnosis fit better. For instance, three presented with EVS, with primary diagnoses of BPPV, Ménière’s disease, and VM, respectively. One patient had a structural lesion (vestibular schwannoma). All three with a final diagnosis of presbyvestibulopathy had additional comorbidities associated with dizziness—most commonly anxiety and polypharmacy—and two experienced improvement in dizziness with antidepressant therapy. Representative vHIT traces for the three patients with presbyvestibulopathy are seen in Figures 3A–C.

**Table 3:** Primary diagnoses and comorbidities of the patients with mild bilateral vestibular hypofunction (bilateral VOR gain 0.6–0.8).

Age	VOR gain		Syndrome	Primary diagnosis	Comorbidities	Follow-up at 1 month
	Right	Left				
75	0.73	0.79	EVS	BPPV	-	No dizziness
84	0.64	0.79	EVS	Ménière’s disease (of the right ear)	-	Episodic vertigo (1/month)
71	0.78	0.71	CVS	Presbyvestibulopathy	Anxiety, polypharmacy	No significant change
83	0.63	0.78	CVS	Presbyvestibulopathy	Anxiety, polypharmacy, sleep disturbance	Dizziness improved by antidepressants
69	0.76	0.78	CVS	Presbyvestibulopathy	Anxiety, migraine	Dizziness improved by antidepressants
62	0.60	0.64	CVS	Vestibular schwannoma	-	No significant change
82	0.67	0.69	EVS	VM	-	Dizziness improved by migraine preventive medications

EVS, episodic vestibular syndrome; CVS, chronic vestibular syndrome.

Seven patients with chronic vestibular syndrome had indeterminate diagnoses despite a comprehensive review of their clinical information (Table 4). Five such patients were aged ≥ 80 years. Most of these patients (*n* = 5, 71.4%) had multiple contributing factors for dizziness. Two patients (28.6%) had UVH of unknown origin (i.e., there was no prior history of an acute vestibular insult). One patient had > 50% carotid stenosis, and another, who presented with vascular parkinsonism, showed predominant cerebral small vessel disease on MRI. Representative vHIT traces for the 88 year-old patient with chronic vestibular syndrome (uncertain primary diagnosis) are seen in Figure 3D.

**Table 4:** The VOR gains and comorbidities of the patients with chronic vestibular syndrome and uncertain diagnoses.

Age	VOR gain		Comorbidities	Follow-up at 1 month
	Right	Left		
73	0.89	0.94	Dementia	No significant change
79	0.85	0.96	Old pontine stroke, atrial fibrillation, DM	No significant change
82	0.82	0.82	Anxiety, carotid stenosis with stenting, arrhythmia with pacemaker	Dizziness improved by antidepressants
82	0.99	0.89	Carotid stenosis (61%), polypharmacy, DM	Dizziness improved without medications
85	0.90	1.15	Cerebral small vessel disease	No significant change
84	0.81	0.71	Mild unilateral vestibular hypofunction, anxiety, orthostatic hypotension, polypharmacy	Dizziness improved by meclizine
88	0.82	0.64	Mild unilateral vestibular hypofunction, anemia	No significant change

DM, diabetes mellitus.

## Discussion

Our study has several important findings:

- VOR gain by vHIT seems to decline with age in patients with symptomatic dizziness; however, most patients aged ≥ 60 (68.6%) still demonstrated normal VOR gains.
- While VM, BPPV, and PPPD were the leading diagnoses in the 60–69 and 70–79 age groups, bilateral vestibulopathy became a common diagnosis among patients aged ≥80, suggesting that vestibular degeneration may present clinical symptoms in this age group.
- In our cohort, however, only three patients (2.9%) met the diagnostic criteria for presbyvestibulopathy, and the prevalence was similar across all three age groups. Moreover, all three patients with presbyvestibulopathy had comorbidities such as anxiety that may have contributed to dizziness. Notably, two of them experienced improvement in dizziness after treatment with antidepressants. Therefore, the association between dizziness and mild BVH remains uncertain. If patients have other treatable conditions, our results suggest that these should be fully addressed.
- Seven patients with chronic dizziness still had indeterminate diagnoses. Their VOR gains were in the normal range (or mildly reduced on only one side), and their dizziness appeared to be multifactorial.

Research on the clinical relevance of presbyvestibulopathy remains limited. A recent study of 707 patients aged ≥ 60 years with chronic vertigo or dizziness found that the vast majority of patients had a better vestibular, neurologic, cardiac, or psychiatric explanation for their dizziness. Only 32 (4.5%) were ultimately found to have presbyvestibulopathy (32). Furthermore, the majority of these 32 patients had comorbidities involving other sensorimotor systems, and truly “isolated” presbyvestibulopathy was extremely rare (0.14%). In another study of 1,218 older patients with dizziness and imbalance, only 33 (2.7%) were diagnosed with presbyvestibulopathy (33). Similarly, in our cohort, just 2.9% of patients met the criteria, and most of these patients also had comorbidities, such as anxiety. One explanation for the low prevalence is that the mild vestibular hypofunction defined in the criteria may not, by itself, cause noticeable dizziness or unsteadiness. Symptoms may only appear when additional factors, such as multisensory deficits or anxiety, are present. Nevertheless, it remains difficult to estimate the extent to which mild vestibular hypofunction contributes to dizziness. Interestingly, in our cohort, two patients with mild BVH presented with EVS rather than CVS; they were ultimately diagnosed with BPPV and Ménière’s disease, respectively. This suggests that mild vestibular hypofunction can also be an incidental finding unrelated to the presenting symptoms.

We therefore suggest that the current criteria for presbyvestibulopathy provide limited clinical utility and may require refinement. For example, in our patients aged ≥ 80 years, up to 13.8% met the criteria for bilateral vestibulopathy (VOR gain < 0.6 bilaterally) without identifiable causes. These cases of idiopathic bilateral vestibulopathy may simply reflect age-related degeneration and should be considered within the framework of presbyvestibulopathy. Furthermore, because the aging process is not always symmetric (34), asymmetric vestibular hypofunction (i.e., asymmetric BVH or UVH) presenting with CVS without other identifiable causes in the elderly should also be considered for inclusion in the diagnostic criteria.

Dizziness in the elderly is increasingly recognized as a geriatric syndrome, reflecting the fact that multiple factors, rather than a single clear etiology, often contribute to symptoms. Epidemiological studies on dizziness in the elderly have shown associations with anxiety and depression, blood pressure fluctuations, cardiovascular disease, polypharmacy, diabetes, visual impairment, and gait disturbance (18, 19). In our study, most patients aged 60–80 had dizziness which could be attributed to common vestibular disorders such as VM, BPPV, and PPPD. These patients (who match a clear primary vestibular diagnosis) should be managed similarly to younger patients with dizziness, albeit with added attention to other *secondary* contributors (blood pressure fluctuations, polypharmacy, visual impairment, cognition, etc.) more prevalent in older adults. In contrast, among patients aged ≥ 80 years, 13.8% had bilateral vestibulopathy and 17.2% had indeterminate diagnoses. In this older age group (age ≥ 80 years), vestibular degeneration and additional contributing factors, such as cerebrovascular disease and polypharmacy, warrant even closer consideration.

In recent years, growing evidence has suggested that unexplained dizziness in the elderly may be associated with cerebral small vessel disease. For example, in a study of 122 older patients, the frequency of severe white matter hyperintensities (Fazekas grade 3) was higher in those with unexplained dizziness than in those with an identifiable cause (20). Another study reported that, compared with asymptomatic controls, patients with unexplained dizziness showed more extensive frontal white matter hyperintensities, as well as reduced white matter integrity in the genu of the corpus callosum and the right inferior longitudinal fasciculus (35). In our own cohort of patients with dizziness of indeterminate cause, one individual presented with lower-body parkinsonism, and his brain MRI indeed demonstrated small vessel disease. However, because cerebral small vessel disease is also commonly found in asymptomatic individuals (36), it remains difficult to establish a causal relationship between dizziness and small vessel disease in individual cases. In addition, because the neck muscles and tendons contain abundant proprioceptive fibers related to head position, orientation, and balance, the potential contribution of degenerative cervical spine disease to dizziness in older adults has been debated for decades (37–40). A recent consensus document from the Bárány Society concluded that current evidence supporting a causal relationship between dizziness and cervical spondylosis remains insufficient, and that further studies are warranted (41).

There are several limitations to this study. First, this was a single-center, retrospective study with a limited sample size; future studies should adopt a multicenter, prospective design with larger cohorts. Second, healthy control participants were not included. Comparing the prevalence of mild BVH between older adults with and without dizziness would provide additional insight. Third, caloric testing and rotational chair testing were not performed, which may have led to an underestimation of the prevalence of presbyvestibulopathy, as these assessments are included in the ICVD criteria (14). Fourth, we only conducted horizontal-canal vHIT. Although the criteria for presbyvestibulopathy do not specify whether vertical-canal vHIT is required, including it may provide a more comprehensive assessment of age-related vestibular decline. Finally, vestibular evoked myogenic potentials (VEMP) were not performed in this study. Although VEMP findings are not included in the current diagnostic criteria for presbyvestibulopathy, age-related decline in VEMP amplitude has been demonstrated in previous studies (42, 43).

In conclusion, although our findings are consistent with previous studies demonstrating age-related declines in vestibular function, we found that the most common diagnoses in dizzy patients aged 60–80 years were broadly the same as those typically seen in younger patients (i.e., VM, BPPV, and PPPD). We therefore suggest that clinicians approach patients in this age range in the same way as younger individuals with dizziness, at least initially. If no apparent primary vestibular or medical diagnosis is found, mild vestibular hypofunction could be one contributor. The increased prevalence of bilateral vestibulopathy among those aged ≥ 80 years indicates that age-related vestibular decline may become clinically relevant issue at this stage and should be considered an important differential diagnosis. That said, the prevalence was still only 14% in those over age 80. Only a small proportion of patients across all age groups met the criteria for presbyvestibulopathy based on vHIT criteria. This finding suggests that the current ICVD criteria for presbyvestibulopathy may not adequately capture the clinical syndrome of the aging vestibular system and may warrant refinement.

## Data Availability

The raw data supporting the conclusions of this article will be made available by the authors, without undue reservation.
