# Supplementary material for: Presbyvestibulopathy: an uncommon cause of dizziness in the elderly
Source: Front Neurol. 2026 Mar 5;17:1744462. doi: 10.3389/fneur.2026.1744462 (PMC12999415; doi:10.3389/fneur.2026.1744462)
Supplement: Supplementary file 1 [file Table_1.docx]

**Supplementary Material**

**“Presbyvestibulopathy: an uncommon cause of dizziness in the elderly”**

**Appendix:** Definitions of dizziness-associated disorders not included in International Classification of Vestibular Disorders from the Bárány Society:

- **Sudden sensorineural hearing loss with vertigo**: acute sensorineural hearing loss ≥30 dB across at least three contiguous frequencies within 72 hours, accompanied by vertigo (1).
- **Light/heavy cupula syndrome**: persistent geotropic or apogeotropic nystagmus on positional testing without other identifiable etiology (2).
- **Post-concussion dizziness**: dizziness with a strong temporal association to head trauma, not attributable to common vestibular disorders.
- **Benign recurrent vertigo**: spontaneous episodic vertigo without auditory or neurological symptoms.
- **Anemia**: diagnosed by laboratory findings.
- **Central vestibulopathy**: diagnosed by brain MRI.

**References:**

1. Stachler RJ, Chandrasekhar SS, Archer SM, Rosenfeld RM, Schwartz SR, Barrs DM, et al. Clinical Practice Guideline: Sudden Hearing Loss. *Otolaryngol Head Neck Surg* (2012) 146(3 Suppl):S1-35. doi: 10.1177/0194599812436449.

2. Hiruma K, Numata T. Positional Nystagmus Showing Neutral Points. *ORL J Otorhinolaryngol Relat Spec* (2004) 66(1):46-50. doi: 10.1159/000077234.

**Table. The primary diagnoses of 102 dizzy patients aged ≥ 60 years**

| **Primary Diagnosis** | **Number** | **Percentage (%)** |
| --- | --- | --- |
| Anemia | 1 | 1.0 |
| Bilateral vestibulopathy | 4 | 3.9 |
| BPPV | 20 | 19.6 |
| Benign recurrent vertigo | 6 | 5.9 |
| Central vestibulopathy | 4 | 3.9 |
| Ménière’s disease | 8 | 7.8 |
| MdDS | 1 | 1.0 |
| Postconcussion dizziness | 2 | 2.0 |
| PPPD | 15 | 14.7 |
| **Presbyvestibulopathy** | **3** | **2.9** |
| Vestibular schwannoma | 1 | 1.0 |
| Vestibular migraine | 21 | 20.6 |
| Vestibular neuritis | 2 | 2.0 |
| Vestibular paroxysmia | 1 | 1.0 |
| SSNHL with vertigo | 1 | 1.0 |
| Heavy cupula syndrome | 1 | 1.0 |
| Indeterminate diagnosis | 11 | 10.8 |
| Total | 102 | 100.0 |

BPPV, benign paroxysmal positional vertigo; MdDS, Mal de débarquement syndrome; PPPD, persistent postural-perceptual dizziness; SSNHL, sudden sensorineural hearing loss.
